# A novel automatic cough frequency monitoring system combining a triaxial accelerometer and a stretchable strain sensor

**DOI:** 10.1038/s41598-021-89457-0

**Published:** 2021-05-11

**Authors:** Takehiro Otoshi, Tatsuya Nagano, Shintaro Izumi, Daisuke Hazama, Naoko Katsurada, Masatsugu Yamamoto, Motoko Tachihara, Kazuyuki Kobayashi, Yoshihiro Nishimura

**Affiliations:** 1 Division of Respiratory Medicine, Department of Internal Medicine, Kobe University Graduate School of Medicine, 7-5-1 Kusunokicho, Chuo-ku, Kobe, Hyogo 650-0017 Japan; 2 Graduate School of System Informatics, Kobe University, 1-1-Rokkodaicho. Nada-ku, Kobe, Hyogo 657-0013 Japan

**Keywords:** Respiration, Engineering

## Abstract

Objective evaluations of cough frequency are considered important for assessing the clinical state of patients with respiratory diseases. However, cough monitors with audio recordings are rarely used in clinical settings. Issues regarding privacy and background noise with audio recordings are barriers to the wide use of these monitors; to solve these problems, we developed a novel automatic cough frequency monitoring system combining a triaxial accelerator and a stretchable strain sensor. Eleven healthy adult volunteers and 10 adult patients with cough were enrolled. The participants wore two devices for 30 min for the cough measurements. An accelerator was attached to the epigastric region, and a stretchable strain sensor was worn around their neck. When the subjects coughed, these devices displayed specific waveforms. The data from all the participants were categorized into a training dataset and a test dataset. Using a variational autoencoder, a machine learning algorithm with deep learning, the components of the test dataset were automatically judged as being a “cough unit” or “non-cough unit”. The sensitivity and specificity in detecting coughs were 92% and 96%, respectively. Our cough monitoring system has the potential to be widely used in clinical settings without any concerns regarding privacy or background noise.

## Introduction

Cough is considered the most common reason for hospital visits, and it has been reported that the annual number of visits for cough is approximately 24 million in the United States^1^. Cough is categorized according to the duration of symptoms: acute cough (0–2 weeks), subacute cough (3–7 weeks) and chronic cough (more than 8 weeks)^2^. In a previous study, we revealed that each type of cough occurred in 19%, 38% and 43% of all the patients who visited our hospitals for cough (n = 207)^3^. Cough greatly impacts our daily lives. Approximately half of patients with chronic cough require frequent hospital visits, and they tend to be depressive or frustrated^4,5^. Additionally, patients with cough consume vast amounts of cough suppressants, despite there being a lack of evidence of their effectiveness^6^. Although empiric therapy is suggested in several guidelines for the management of chronic cough^7^, it has been reported that half of patients with chronic cough do not receive definite diagnoses, even after visiting their doctors many times^8^. Considering these facts, to reduce the inappropriate use of medications for cough and to assess the clinical state of patients with cough, there is a strong need for an index that can precisely assess cases of cough.

Currently, there are some subjective tools that have been validated for assessing cough: the cough visual analog scale and cough-specific quality of life questionnaires^9^. Although these methods correspond well with patients’ perceptions of the severity of cough, they are sometimes unreliable because they can be influenced by patients’ mood, consciousness or recall bias^10^. Therefore, methods that can objectively assess cough in terms of its frequency or intensity are essential.

One of the most commonly used cough frequency monitors that is already available is the Leicester cough monitor (LCM), which records sounds continuously from a microphone with a digital sound recorder^11^. It has a cough frequency measurement system that uses an automated cough detection algorithm. LCM has been used in some cough studies^12–14^, but it is rarely used during treatment of patients with cough. Although this type of cough monitor with audio recordings has been validated in research settings, we think that there are two major problems when it is used for clinical purposes. First, on the basis of audio recordings only, is it difficult to distinguish patients’ cough sounds from other environmental sounds (especially someone else’s cough). The second problem is that privacy issues arise when voice recorders are used, especially regarding patients’ private conversations. Therefore, patients may hesitate to use the monitor due to privacy concerns.

Therefore, in this study, we developed a new type of automatic cough frequency monitoring system that does not involve audio recordings. Specifically, we established a new cough frequency monitoring system that combines a triaxial accelerometer and a wearable stretchable strain sensor.

## Results

### Subject characteristics

The characteristics of the subjects in this study are summarized in Supplementary Table 1. All the healthy volunteers were never smokers and did not have any respiratory diseases. Among the 10 patients with cough, 8 were ex- or current smokers. At study enrollment, 6 patients had previously been diagnosed with chronic obstructive pulmonary disease. We retrieved spirometry test results from only 6 patients (5 ex-smokers and 1 never-smoker) because spirometry tests were prohibited in our hospital for a while due to the COVID-19 pandemic.

### The accuracy of our monitoring system for assessing cough frequency

The sensitivity and specificity rates of our monitoring system among the healthy volunteers (n = 11), the patients with cough (n = 10), and all participants (n = 21) are shown in Tables 1, 2 and 3. The sensitivity and specificity were 92% and 96%, respectively among all participants (Table 3).

**Table 1:** Sensitivity and specificity of our cough monitoring system in healthy volunteers (n = 11).

Subject no.	Actual number of cough units	Sensitivity (%)	Specificity (%)	PPV (%)	NPV (%)
1	7	86	96	75	98
2	6	100	81	55	100
3	3	100	100	100	100
4	5	100	100	100	100
5	3	100	100	100	100
6	4	100	100	100	100
7	3	100	67	75	100
8	5	100	100	100	100
9	5	80	100	100	97
10	2	100	91	50	100
11	5	80	98	80	98
Total	48	94	95	80	99

All healthy volunteers were asked to cough voluntarily.

*NPV* negative predictive value, *PPV* positive predictive value.

**Table 2:** Sensitivity and specificity of our cough monitoring system in patients with cough (n = 10).

Subject no.	Actual number of cough units	Sensitivity (%)	Specificity (%)	PPV (%)	NPV (%)
1	19	100	100	100	100
2	10	80	100	100	50
3	3	33	100	100	94
4	2	100	100	100	100
5	3	100	100	100	100
6	3	100	75	75	100
7	2	100	75	67	100
8	5	100	83	83	100
9	3	100	98	75	100
10	4	75	100	100	93
Total	54	91	97	92	97

*NPV* negative predictive value, *PPV* positive predictive value.

**Table 3:** Sensitivity and specificity of our cough monitoring system in all participants (n = 21).

	Actual number of cough units	Sensitivity (%)	Specificity (%)	PPV (%)	NPV (%)
Total	102	92	96	86	98

*NPV* negative predictive value, *PPV* positive predictive value.

### The utility of a triaxial accelerometer for assessing cough frequency

While it was revealed that our monitoring system, which used both a triaxial accelerometer and a stretchable strain sensor, showed high sensitivity and specificity in assessing cough frequency (Table 3), we specifically examined the utility of a triaxial accelerometer among all participants (n = 21). The sensitivity and specificity of using an accelerometer without a stretchable strain sensor were 91% and 95%, respectively. Therefore, the accuracy improved slightly when the accelerometer was combined with a stretchable strain sensor in this study.

### The effects of exercise on the utility of the cough monitoring system

Because the subjects sat on a chair while coughs were measured in this study, it was necessary to investigate the effects of exercise on the utility of this cough monitoring system. Therefore, one healthy volunteer was asked to walk for one minute while wearing our cough frequency monitor (an accelerometer and a strain sensor) (Exercise 1). He was also asked to repeatedly stand and sit every 5 s for one minute with the cough monitor (Exercise 2). During these exercises, he was not allowed to cough. Then, we examined the frequency with which the units produced by the exercises were mistakenly judged as coughs. The data obtained from these exercises are shown in Supplementary Data 1.

The total number of units produced by the two exercises was 24 (because each 1-min dataset was divided into units of 5 s). When these 24 units were used as the test dataset and labeled by the existing algorithm mentioned above, only 3 units (13%) were mistakenly judged as cough units. Moreover, when we added the former or latter half of the 24 units to the existing training dataset and tested the other half of the units, all the tested units were correctly labeled as non-cough units. In summary, there are likely no severe effects of exercise on the utility of our cough monitoring system.

### Generalizability of the current cough monitoring system

Finally, we examined the generalizability of the current cough monitoring system. In other words, we investigated whether the training dataset in this study was sufficient for testing a completely new patient (dataset). To test this hypothesis, datasets from all participants except one (n = 20) were selected, and 60% of all units with amplitudes greater than the threshold were included in the training dataset. Then, based on this training dataset, all the units of the excluded patient (n = 1) with amplitudes greater than the threshold were tested. We repeated this analysis 21 times and examined the sensitivity and specificity. In total, 275 cough units and 891 non-cough units were included in the test dataset, and the sensitivity and specificity were relatively high, as they were 85% and 93%, respectively. This result suggests that the training dataset used for our cough monitoring system is sufficient for testing the dataset of a completely new subject.

## Discussion

In recent years, it has been recognized that objective evaluations of cough are vital, and in some previous studies, cough frequency has been shown to be an important factor for monitoring the clinical state of patients with respiratory diseases, such as asthma and chronic obstructive pulmonary disease^13,15–17^. In these kinds of studies, cough monitors with audio recordings are generally used. However, curiously, these cough monitors are rarely used in clinical settings, and we think that issues regarding privacy and background noise are barriers to the wide use of these monitors in clinical settings.

In this study, by combining a triaxial accelerometer and a stretchable strain sensor, we developed a new type of automatic cough frequency monitoring system that has high sensitivity and specificity. While cough monitors with audio recordings have some specific problems (regarding privacy and background noise) in clinical settings, the present system that does not use audio recordings can solve these problems. Our cough monitor is also suitable for ambulatory settings because the devices are small and light. Moreover, it may be possible to automatically assess cough intensity by calculating the amplitudes of waveforms displayed by our cough monitor. Although it is thought that cough intensity is as important as cough frequency with respect to its impact on quality of life, there is still no portable monitor that has been validated for assessing cough intensity^18^. In future studies, we would like to confirm that our cough monitor is useful not only for measuring cough frequency but also for measuring cough intensity.

This is the first cough monitor using a strain sensor. In the current study, it was revealed that the use of a triaxial accelerometer only yields a sensitivity and specificity of 91% and 95%, respectively, in assessing cough frequency. However, the accuracy of the cough monitor improved slightly when the accelerometer was combined with a strain sensor (sensitivity 92%, specificity 96%). Recently, small, flexible, and stretchable strain sensors have been recognized as useful tools for human motion monitoring^19^. Additionally, with technological advances, it is expected that increasingly more small, convenient sensors for human motion monitoring will be available. According to the results of the present study, a cough monitor with better performance can be created by combining an accelerometer with another biometric sensor. Additionally, the results of this study suggest that the combination of motions of two different parts of the body (for example, the epigastric region and neck in this study) can be used to detect coughs, with high accuracy. On the other hand, body temperature is a basic physiological parameter of patients’ condition. So, we think that incorporating temperature sensors into our system is also very useful when monitoring coughs of patients with fever (such as pneumonia), although it is necessary to verify the accuracy of the non-invasive estimation of deep body temperature. To develop the best cough monitor, more investigations are needed to verify the optimal combination of biometric sensors and optimal placement of the sensors on the body.

In this study, we have shown that our novel cough monitoring system is promising and easy to use in clinical settings for the assessment of cough frequency. Moreover, it was proven that there are no severe effects of exercise on the utility of the present cough monitoring system. However, the present results are still preliminary, and studies with more subjects (various age groups and ethnic groups) and longer measurement times (24 h) in real-world situations are required. Moreover, our algorithm may underestimate the actual number of coughs. For example, 10 sequential coughs during 10 s were counted as 2 cough units by our algorithm, because measurement data from our cough monitoring system were divided into consecutive small units lasting 5 s each. Therefore, it is necessary to investigate whether this feature of our algorithm has any impact on the evaluation of cough frequency. We also need to verify whether our cough monitoring system can accurately recognize coughs of patients with other respiratory diseases such as COVID-19 or asthma. However, our algorithm can handle more complex clustering, and we can expect that the availability of more training datasets from more subjects with various respiratory diseases will improve the accuracy of our cough frequency monitoring system, which uses a deep-learning-based algorithm. Also, in further studies, we have to confirm that cough frequency measured by our system correlates well with the result of cough-specific quality of life questionnaires.

In summary, we developed a novel cough monitoring system combining an accelerator and a stretchable strain sensor. This system that does not involve audio recordings has the potential to be widely used in clinical settings without any concerns regarding privacy or background noise.

## Methods

### Ethical issues

This study was performed at Kobe University Hospital. “Kobe University Hospital Clinical and Translational Research Center” ethics committee has approved the study (permission number: 300034). Written informed consent was obtained from all subjects included in the study. All procedures performed were in accordance with the ethical standards of the institutional and national research committee and with the 1964 Helsinki declaration and its later amendments or comparable ethical standards.

### Devices

**Figure 1:**
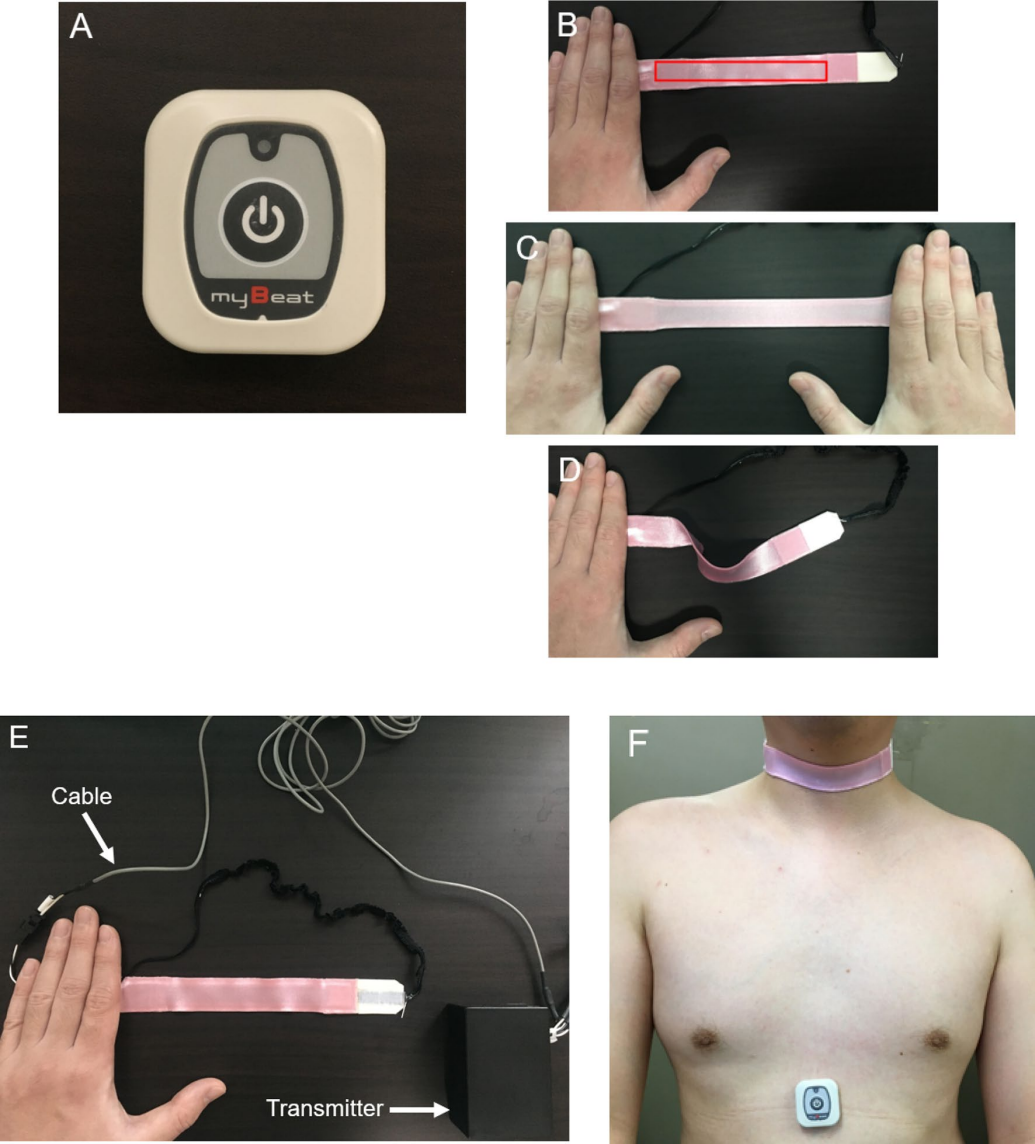
Devices for cough frequency monitoring system in this study. (**A**) Triaxial accelerometer (WHS-3 SENSOR). (**B**–**D**) Stretchable strain sensor (C-STRETCH). There is a sensor in the red square (**B**). This sensor is soft and can be easily stretched (**C**,**D**). (**E**) Strain sensor, cable, and transmitter. (**F**) The triaxial accelerometer was attached to the epigastric region. A stretchable strain sensor was worn around the participant’s neck.

In this study, we used two kinds of devices that do not interfere with each other. One device is a sensitive triaxial accelerometer (WHS-3 SENSOR, UNION TOOL CO., Tokyo, Japan). This accelerometer can record acceleration signals in three orthogonal directions from the area at which it is attached every 31.25 ms (Fig. 1A). In this study, to detect coughs, it was attached to the epigastric region. The accelerometer detects movements of each participant’s epigastric region by recording its horizontal (X), vertical (Y), and longitudinal (Z) positions. The other device is a wearable stretchable strain sensor (C-STRETCH, Bando Chemical Industries Ltd., Hyogo, Japan) (Fig. 1B–E). Its mechanism was described well in another study using this sensor^19^. In brief, each sensor has elastomer layers and electrode layers. With this parallel plate structure, each sensor acts as a capacitor. The detection area of this sensor can extend to almost double its size, and the capacitance of this sensor is linearly related to the strain of the sensing area. This sensor can sensitively detect the expansion of the skin. In this study, the participants wore the stretchable strain sensor around their neck, and the expansion of throat skin was evaluated by this sensor. Both the accelerometer and strain sensor are small, light, and suitable for ambulatory use. These devices were worn simultaneously (Fig. 1F). Data from these devices were transferred wirelessly to tablets or personal computers. We confirmed that no coughs were induced by wearing these devices for 30 min among 4 healthy volunteers (2 males and 2 females).

### Cough frequency measurements

In this study, from September 2019 to June 2020, 11 healthy adult volunteers with no symptoms of cough and 10 adult patients who had symptoms of cough were consecutively enrolled. For the cough frequency measurements, the participants were equipped with two devices (a triaxial accelerometer and a stretchable strain sensor) for 30 min, while they sat on a chair in a room. While sitting, they were allowed to talk and move their body. The healthy volunteers were asked to cough voluntarily. For the entire duration of measurement, a researcher observed each participant from the same room and manually recorded and counted the coughs.

### Waveforms by cough monitoring system

**Figure 2:**
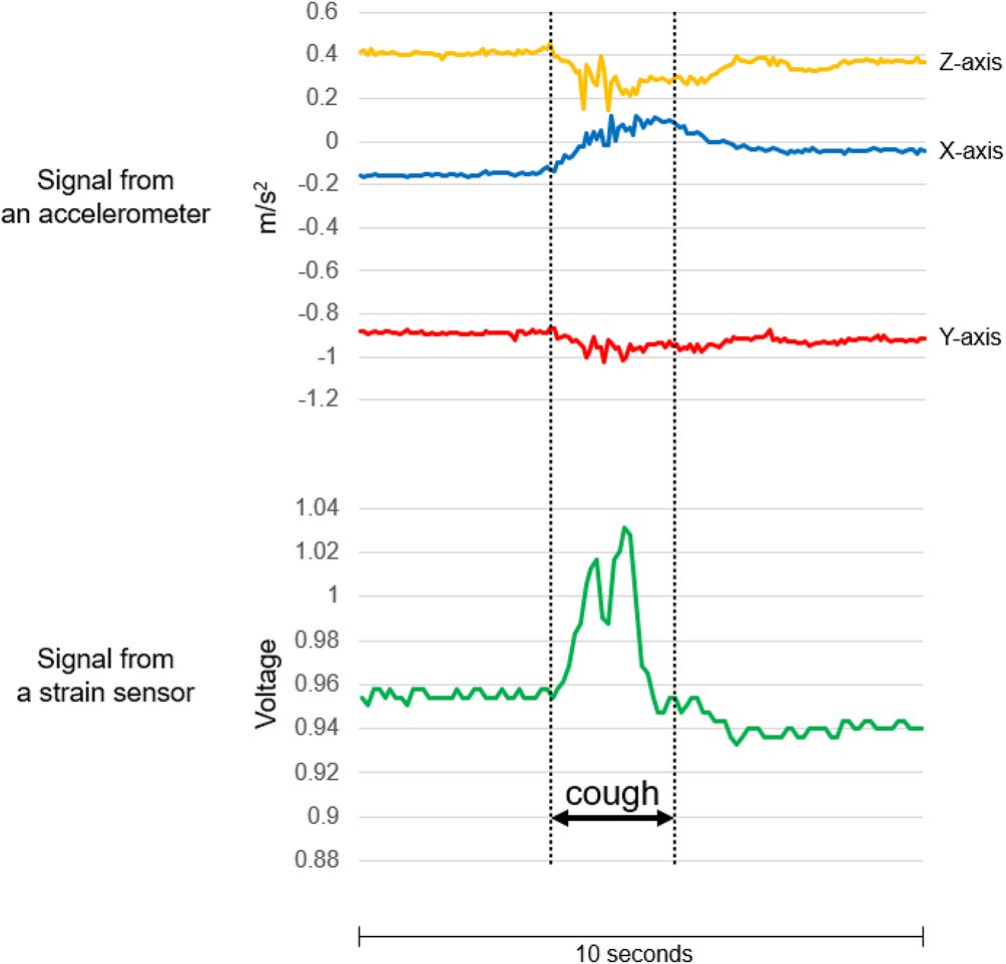
Typical cough waveforms from a triaxial accelerometer (WHS-3 SENSOR) and a strain sensor (C-STRETCH) in a subject in this study.

**Figure 3:**
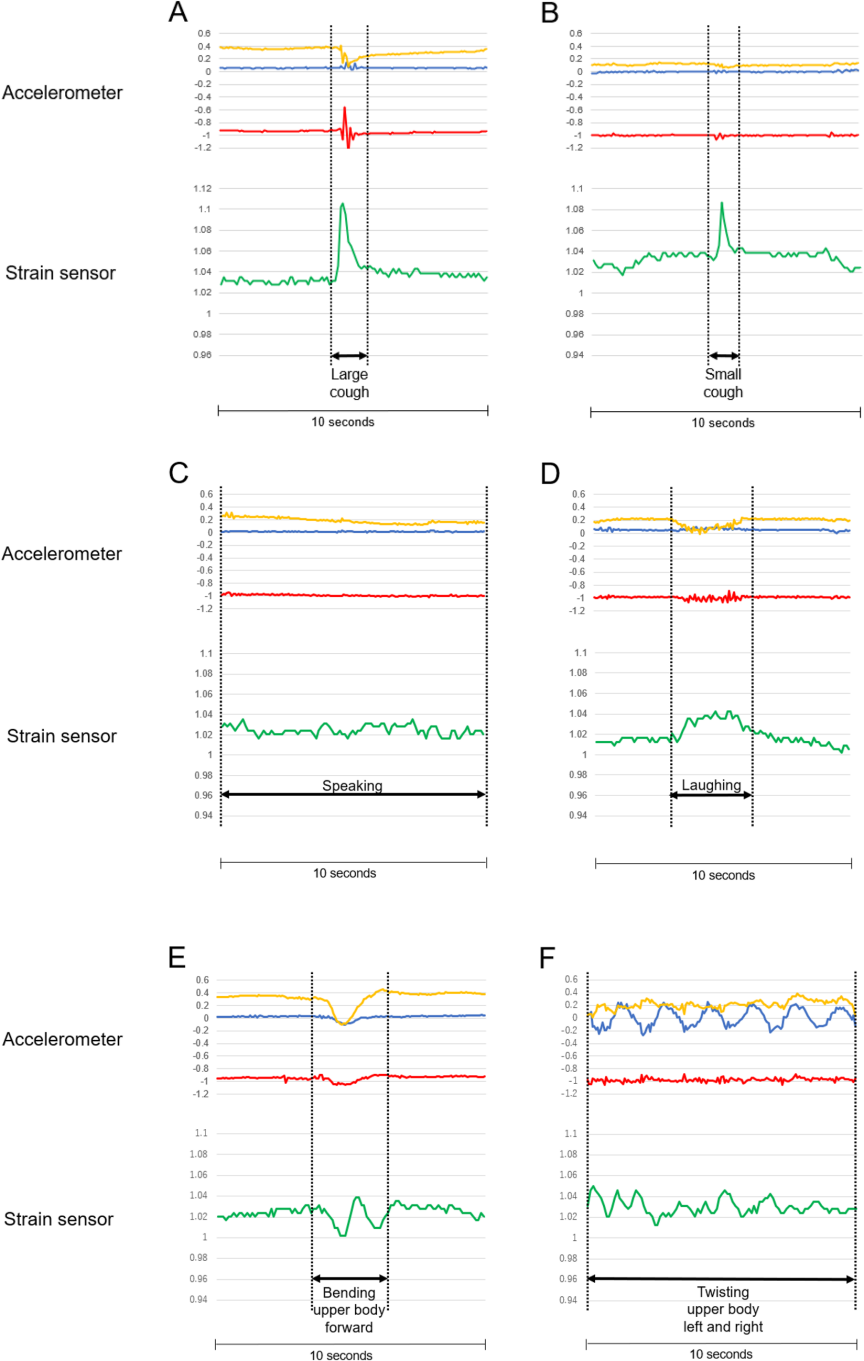
Waveforms classified by cough intensity, conversation, and upper body movements in a subject in this study. (**A**,**B**) Waveforms of a large cough and a small cough. (**C**,**D**) Waveforms produced by speaking and laughing. (**E**) Waveforms produced by the subject leaning his or her upper body forward and returning to a neutral position while sitting. (**F**) Waveforms produced by the individual twisted his or her upper body to the left and right while sitting.

When the subjects coughed, the two different devices (the triaxial accelerometer and stretchable strain sensor) displayed specific waveforms. Typical cough waveforms are shown in Fig. 2. The positive and negative deflections of an accelerometer represent the vectors in opposite directions on each axis. Cough intensity (large cough or small cough) is represented by the wave height (Fig. 3A,B). The cough waveforms were distinguishable from those produced by speaking and laughing (Fig. 3C,D). The cough waveforms were also distinguishable from those produced by upper body movements (Fig. 3E,F).

### Cough frequency monitoring algorithm

For the development of the automatic cough frequency monitoring algorithm, the participants’ measurement data from the triaxial accelerometer and stretchable strain sensor were divided into consecutive small “units” lasting 5 s each. We defined “cough units” as those corresponding to when a subject coughed within the 5-s period. We defined “non-cough units” as those corresponding to when a subject did not cough within the 5 s. Whether each unit corresponded to a “cough unit” or “non-cough unit” was determined by the observer who manually counted the cough records. These “labels” were used for the machine learning algorithm.

**Figure 4:**
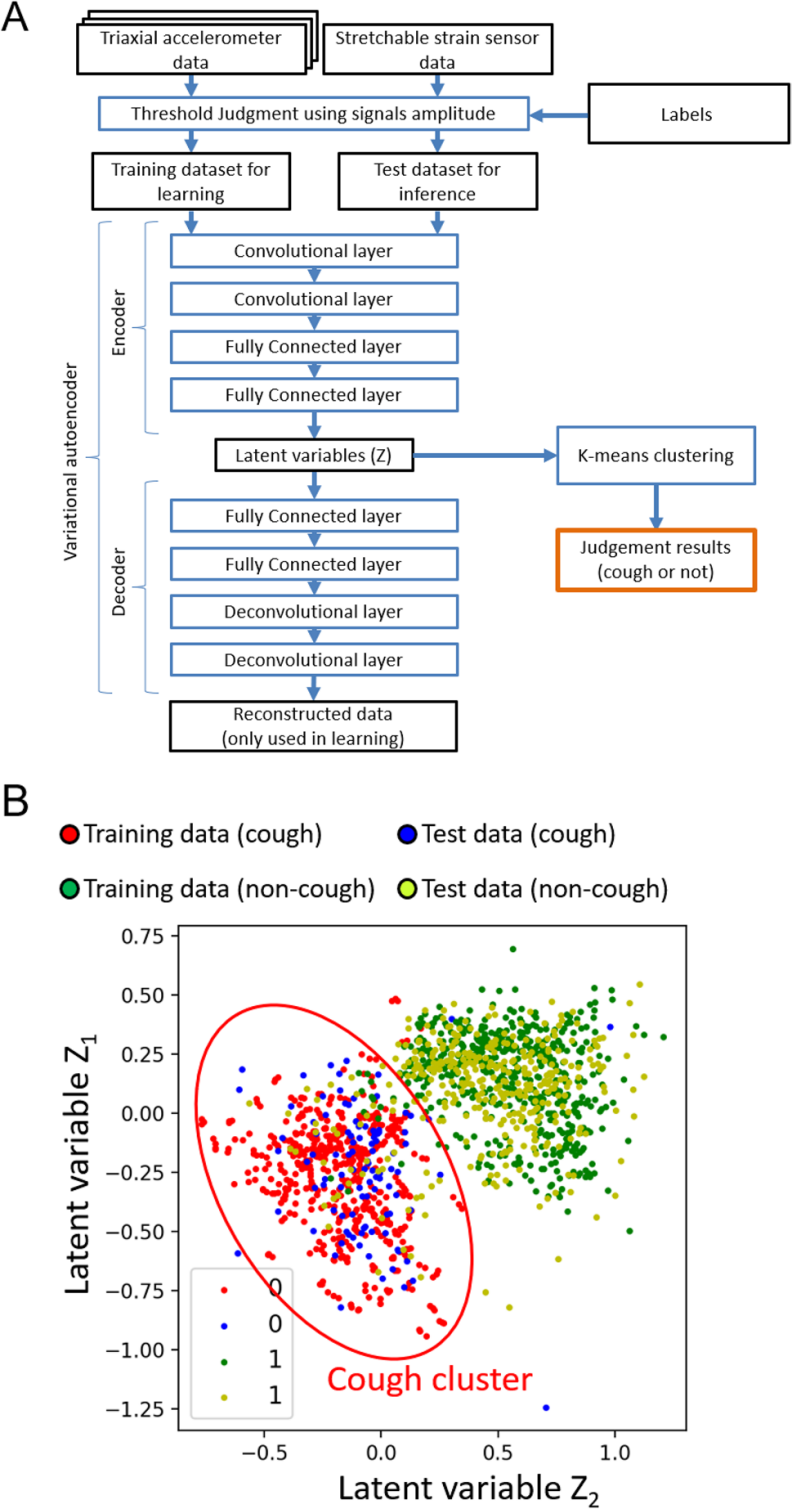
Cough frequency monitoring algorithm used in this study. (**A**) Signal amplitude threshold, structure of variational autoencoder (VAE) and clustering for cough detection. Signal amplitude thresholds were set using data that were determined as coughs by the “labels”, and data units with an amplitude greater than the threshold were categorized into training datasets and test datasets. VAE, a machine learning algorithm with deep learning, consists of a network called an encoder and decoder and can automatically extract and learn multilevel features of coughs in the latent variable space. K-means clustering was used to determine whether the input data units were “cough units” or “non-cough units” from the latent variables. In short, the VAE built a feature extraction network using a training dataset and is clustered by the k-means algorithm. Then, based on the performance of the learned network and clustering results, the test dataset units were automatically labeled as cough or non-cough units. (**B**) Example of a clustering result in this study. Using the training data, the VAE extracted features of cough in the latent variable space (latent variables Z_1_ and Z_2_), and the results were clustered by the k-means algorithm. Based on this algorithm, the area within the red circle was defined as a cough cluster. Our algorithm was created by using Python 3.9 and PyTorch 1.5.1.

A variational autoencoder (VAE), which is a machine learning algorithm using deep learning, was used for cough feature extraction. As shown in Fig. 4A, VAE consists of a network called an encoder and decoder. The encoder compresses the input data unit into a latent variable space, and the decoder restores the input data from the latent variable space. In other words, the VAE can automatically extract and learn multilevel features of coughs in the latent variable space. To determine whether the input data units were “cough units” or “non-cough units” from the latent variables, a k-means clustering algorithm was used. Figure 4B shows an example of the clustering results.

To train and evaluate the VAE and k-means, all measured units were divided into training and test datasets (Fig. 4A). First, signal amplitude thresholds were determined to select the units that may have coughs from all the units from all participants (n = 21). The thresholds were set using the datasets labeled as “cough units”. Next, 60% of the units with an amplitude greater than the threshold were included in the training dataset, and the remaining 40% of the units were used as the test dataset. The VAE built a feature extraction network using the training dataset and was clustered by the k-means algorithm. The performance of the learned network and clustering results were evaluated by the test dataset (Fig. 4A). Our algorithm was created by using Python 3.9 and PyTorch 1.5.1.

### Data presentation and analysis

**Figure 5:**
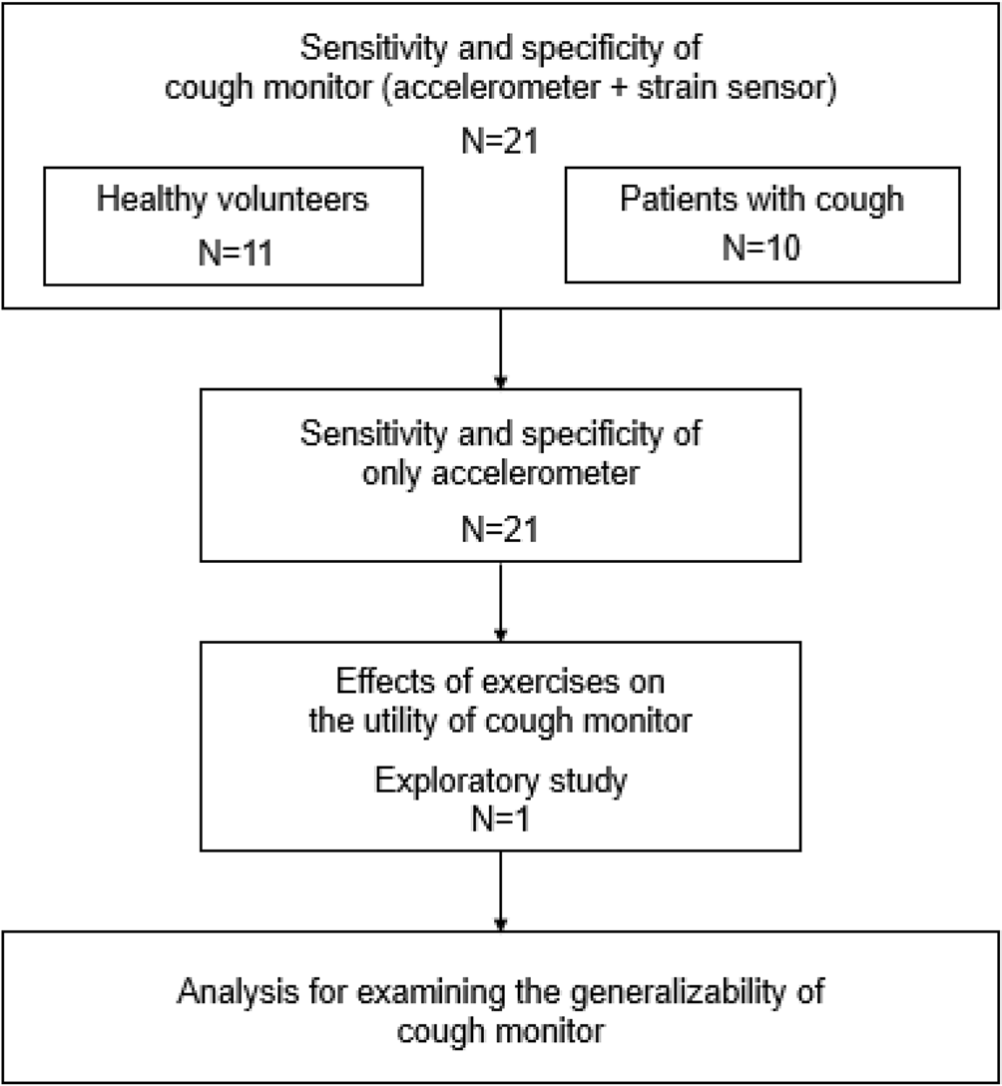
Schema of analyses conducted in this study. First, we evaluated the sensitivity and specificity rates of our cough frequency monitoring system among the healthy volunteers (n = 11), the patients with cough (n = 10), and all participants (n = 21). Second, the sensitivity and specificity of using data from only the accelerometer were examined. Third, the effects of exercise on the utility of our cough monitoring system were exploratorily investigated (n = 1). Finally, we investigated whether the training dataset in this study was sufficient for testing a completely new patient (analysis for examining the generalizability of our cough monitor).

Analyses were carried out using JMP 9.0.2 statistical software (SAS Institute Inc., NC, USA). The sensitivity (the percentage of cough units that were correctly identified by our algorithm) and specificity (the percentage of non-cough units that were correctly identified by our algorithm) were calculated among the healthy volunteers (n = 11), the patients with cough (n = 10), and all the participants (n = 21). The sensitivity and specificity of using only an accelerometer were also calculated. Additionally, the effects of exercise (while wearing the cough monitor, one subject was asked to walk or repeatedly stand and sit) on the results of our algorithm were examined. Finally, we analyzed the generalizability of our system. A schema of the analyses conducted in this study is shown in Fig. 5. The data for the continuous variables are summarized using means (standard deviation).

## Supplementary Information

Supplementary Dataset 1.

Supplementary Table 1.

## Data Availability

The data supporting the results of the present study are available within the Supplementary Data file.
