# Supplementary material for: A novel automatic cough frequency monitoring system combining a triaxial accelerometer and a stretchable strain sensor
Source: Sci Rep. 2021 May 11;11:9973. doi: 10.1038/s41598-021-89457-0 (PMC8113562; doi:10.1038/s41598-021-89457-0)
Supplement: Supplementary file 2 — Supplementary Table 1. [file 41598_2021_89457_MOESM2_ESM.docx]

**Supplementary Table 1** Subject characteristics

|  | Healthy volunteers | Patients with cough |
| --- | --- | --- |
| Subjects, n | 11 | 10 |
| Age, years | 39 (11)^a^ | 76 (6)^a^ |
| Sex (male), n | 6 | 5 |
| FEV_1_ % predicted | - | 80 (21)^a^ (n=6)^b^ |
| FEV_1_/FVC % | - | 67 (17)^a^ (n=6)^b^ |
| FVC % predicted | - | 95 (17)^a^ (n=6)^b^ |
| Never smoker, n | 11 | 2 |
| Ex-smoker, n | 0 | 7 |
| Current smoker, n | 0 | 1 |
| Pack-year history | 0 | 52 (43)^a^ |

^a^ The numbers indicate means (standard deviation)

^b^ The numbers in parentheses indicate the numbers of patients with data available

FEV_1_ forced expiratory volume in one second, FVC forced vital capacity
